# Facilitated Qualitative Determination of Insulin, Its Synthetic Analogs, and C-Peptide in Human Urine by Means of LC–HRMS

**DOI:** 10.3390/metabo11050309

**Published:** 2021-05-12

**Authors:** Andreas Thomas, Lukas Benzenberg, Lia Bally, Mario Thevis

**Affiliations:** 1 Institute of Biochemistry/Center for Preventive Doping Research, German Sport University Cologne, Am Sportpark Müngersdorf 6, 50933 Cologne, Germany; benzenberglukas@gmail.com (L.B.); m.thevis@biochem.dshs-koeln.de (M.T.); 2 Department of Diabetes: Endocrinology, Nutritional Medicine, and Metabolism, Inselspital, Bern University Hospital, University of Bern, 3010 Bern, Switzerland; Lia.Bally@insel.ch; 3 European Monitoring Center for Emerging Doping Agents (EuMoCEDA), 50933 Cologne/Bonn, Germany

**Keywords:** high-resolution mass spectrometry, mixed-mode solid-phase extraction, doping controls

## Abstract

The increasing importance to determine bioactive peptide hormones such as insulin, its synthetic analogs, and C-peptide in urine samples represents an analytical challenge. The physiological concentrations of insulin in urine are commonly found at sub-ng/mL levels and thus represent a complex analytical task. C-peptide concentrations, on the other hand, tend to be in the moderate ng/mL range and are hence much easier to determine. Insulin and C-peptide are important in the diagnostics and management of metabolic disorders such as diabetes mellitus and are also particularly relevant target analytes in professional sports and forensics. All insulins are classified on the World Anti-Doping Agency’s (WADA) list of prohibited substances and methods in sports with a minimum required performance level (MRPL) of 50 pg/mL. Until now, methods combining immunoextraction and subsequent mass spectrometric detection have mostly been used for this purpose. With the method developed here, sample preparation has been simplified considerably and does not require an antibody-based sample purification. This was achieved by a sophisticated mixed-mode solid-phase extraction and subsequent separation with liquid chromatography coupled to high-resolution mass spectrometry. Included target insulins were human, lispro, glulisine, aspart, glargine metabolite, degludec, and additionally, human C-peptide. The method was validated for the synthetic insulin analogs considering WADA requirements including specificity, limit of detection (10–25 pg/mL), limit of identification, recovery (25–100%), robustness, carry over (<2%), and matrix effects. All sample preparation steps were controlled by two stable isotope-labeled internal standards, namely, [[2H10] LeuB6, B11, B15, B17]-insulin and [[13C6] Leu26, 30] C-peptide. Finally, the method was applied to samples from patients with *diabetes mellitus* treated with synthetic insulins.

## 1. Introduction

While insulin is an important, endogenous peptide hormone that regulates blood glucose homeostasis and other aspects of metabolism, the cosecreted C-peptide owns merely limited (if any) or unknown physiological effects. C-peptide and insulin are produced in equimolar amounts from the same single-chain prohormone (proinsulin) in the pancreatic β-cells [1,2]. The single-chain, linear C-peptide consists of 31 amino acids with an isoelectric point (pI) of 2.8. In contrast, the structure of insulin is more complex, because it comprises two peptide chains (α+β-chain) with three disulfide bridges and a total of 51 amino acids (21 α-chain, 30 β-chain) and a pI of 5.3. The amino acid sequences of both hormones (as well as the synthetic analogs) are shown in Table 1. Here, the comparison of the sequences illustrates that the differentiation of the respective analogs is enabled by mass spectrometric approaches by means of their distinct molecular masses and/or their diagnostic product ions. All synthetic insulin analogs have their individual and specific pharmacokinetic profile, which facilitates their usage as therapeutic agents to treat *diabetes mellitus* (DM) [3]. Generally, because C-peptide is physiologically largely inert, it has negligible hepatic clearance and is therefore renally excreted in much higher amounts than insulin. [4] In contrast, insulin has a plasma half-life of only a few minutes and urinary concentrations are accordingly very low. The World Anti-Doping Agency (WADA) has set the minimum required performance level (MRPL) for all insulins to 50 pg/mL, and this concentration represents by far the lowest MRPL of all prohibited substances, emphasizing the analytical challenge for these bioactive peptides [5]. The present method is mainly developed in order to facilitate the analysis of urinary doping control samples for synthetic insulins. All published methods for the determination of insulin and C-peptide in urine by means of liquid chromatography/mass spectrometry mainly use immunoaffinity extraction of the target peptides from the matrix [3,6,7,8,9,10,11,12]. These immunoaffinity-enriched sample aliquots are of highly purified quality and allow for nanoscale liquid chromatography. The specificity achieved by these assays is outstanding, but these methods suffer from laborious and time-consuming sample preparation, thereby precluding higher sample throughput. Therefore, a solid-phase extraction-based assay was desirable to provide an alternative approach that potentially simplifies the urine preparation procedure and allows for greater assay scalability. Noteworthy, those assays were already developed for blood samples recently [13,14]. In the present approach, we aimed at the simultaneous determination of insulin (human, lispro, glulisine, aspart, glargine metabolite, degludec, porcine, and bovine) and C-peptide in urine after sample preparation by protein precipitation and mixed-mode cation exchange solid-phase extraction, followed by detection by liquid chromatography–high-resolution mass spectrometry (LC–HRMS). The long-acting synthetic insulin analog detemir was not included because it is not excreted into urine and only the metabolite DesB30 human insulin is detectable here [15]. The main parameters of the target peptides are summarized in Table 1. Two stable isotopically labeled internal standards [[2H10] LeuB6, B11, B15, B17]-insulin (human) and [[13C6] Leu26, 30]-C-peptide (human) were used to control all sample preparation steps.

## 2. Results and Discussion

### 2.1. Liquid Chromatography/Mass Spectrometry

**Figure 1:**
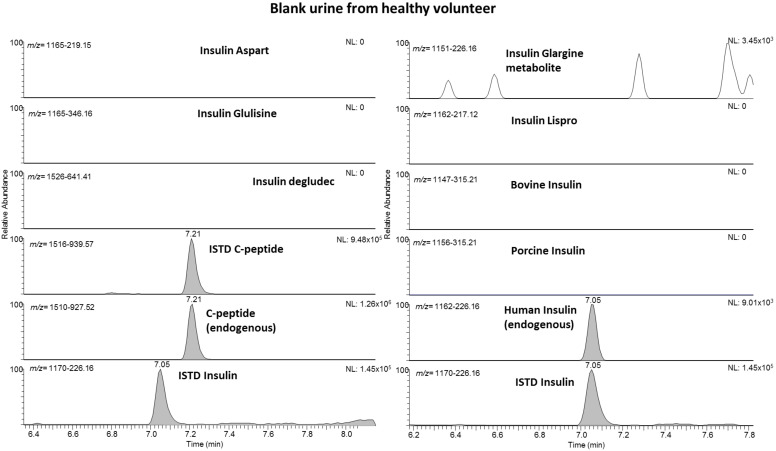
Extracted ion chromatograms of a blank sample from a healthy volunteer showing signals for endogenous human insulin and C-peptide only.

**Figure 2:**
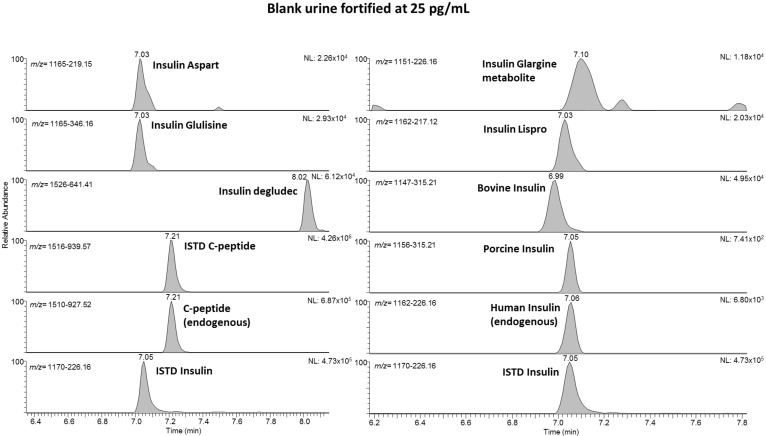
Extracted ion chromatograms of a human blank sample fortified at 50% of the MRPL with 25 pg/mL for all insulin analogs. C-peptide and human insulin are endogenous and were not fortified.

Besides the efficient sample cleanup and concentration by means of the mixed-mode cation exchange, also the liquid chromatographic and mass spectrometric conditions require careful selection in order to enable fulfilling all mandatory criteria. Concerning liquid chromatography, preconcentrating the target peptides on a trapping column before switching and unloading the trapped analytes onto the analytical column represents an effective tool to clean up and concentrate a comparably high injection volume (up to 25 µL), which still contains a comparably high load of inorganic salts and other undesired residues. On the other hand, the employed high-resolution/high-mass accuracy mass spectrometer offers the potential to multiplex and group different precursor ions before collision-induced dissociation. With the knowledge of which product ion is diagnostic to the respective target analyte, the multiplexing enables fast data acquisition rates with a sufficient number of data points over the chromatographic peak. Figure 1 shows example chromatograms of a blank sample from a healthy volunteer; accordingly, a fortified sample (at 25 pg/mL) is illustrated in Figure 2. While only endogenous human insulin and C-peptide are detected in Figure 1, all included target peptides were identified in the fortified sample. The presence of insulin lispro in addition to human insulin (they share the same molecular mass, see Table 1) is detected by the diagnostic product ion at *m/z* 217.12.

### 2.2. Validation

Full method validation was performed as an initial testing procedure according to the requirements of the ISL [16]. The main results are summarized in Table 2. The specificity showed that no interfering signals occur for the synthetic insulins when measuring urine from healthy nontreated volunteers. Here, in all samples, endogenous human insulin and C-peptide were detected (example see Figure 1). The limits of detection ranged at approximately 10–25 pg/mL for all target analytes, which is sufficient according to WADA requirements, where the MRPL is set to 50 pg/mL for all insulin analogs. At this level (50 pg/mL), the respective signal in the chromatogram was detected for all analogs in all six different urine samples with a signal to noise ratio (S/N) of >3. The results for the LOI, determined at 25, 10, and 5 pg/mL (Table 2) show that the synthetic insulins are largely detectable at 25 pg/mL and only barely at 5 pg/mL. Figure 2 shows a typical chromatogram from a sample fortified at 25 pg/mL. Typical recoveries range from 26 to 53% (exemption: C-peptide at 100%) with a relative standard deviation between 2 and 24%. The robustness of the method was evaluated according to the drying time in the heated vacuum centrifuge. Here, it was shown that the target peptides are not suffering from degradation when they are exposed to heat in the vacuum centrifuge for up to 10 min after dryness. This represents important information for preparing large sample sets with different individual drying times. 

Carryover from a highly concentrated sample into the next injection was tested at four times MRPL (200 pg/mL). Here, the maximum carryover for all included target peptides was below 1–2% of the preceding injection. The impact of matrix effects yielded no significant ion enhancement or ion suppression (+/−20% rel. std. deviation) in five different blank samples, compared to a neat standard solution. 

### 2.3. Proof of Concept

**Figure 3:**
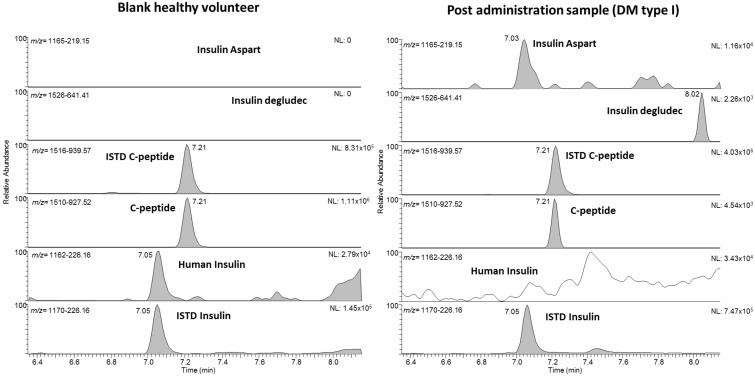
Extracted ion chromatograms of a blank sample from a healthy volunteer (left) with endogenous human insulin and C-peptide only. On the right, a postadministration sample from a patient suffering from *diabetes mellitus* (type I, patient No 5) with a regular treatment of insulin aspart and degludec. Only traces of endogenous C-peptide as well as no endogenous human insulin was detected in this sample.

The applicability of the method to authentic patient samples was verified with five human urine samples from a person with *diabetes mellitus* (DM) receiving synthetic insulin therapy. Figure 3 shows exemplarily the chromatograms from one person using the fast-acting insulin aspart (Novorapid) and the ultralong-acting insulin degludec (Tresiba). Both synthetic insulins were detected in the respective diagnostic product ion trace with signals at 7.0 min for aspart (*m/z* 1166 resp. 1457 to 219.15) and at 8.0 min (*m/z* 1526 to 641) for degludec. The observation that no human insulin and only traces of endogenous human C-peptide were detected support the diagnosis of type 1 diabetes mellitus, which is characterized by an absolute endogenous insulin deficiency. Table 3 shows all the main characteristics and results from the five samples derived from synthetic insulin-treated patients. All expected insulins were detected in respective samples, which further confirms the proof-of-concept for the method. The estimated concentrations of all detected insulins are in the validated working range of the assay.

### 2.4. Limitations of the Assay/Potential Applications 

At the present status of the assays, the data interpretation is focused on qualitative results only, and a reliable comparison to other assays (e.g., LBA) is not possible. For example, in doping controls, already the qualitative determination (and confirmation) of synthetic insulin in a sample from an athlete (nondiabetic without therapeutic use exemption) constitutes an adverse analytical finding [16]. The same scenario would be valid for forensic samples from nondiabetic patients (surreptitious administration, medication errors, etc.). Noteworthy, postmortem urine samples tend to fast degradation of the synthetic as well as the endogenous insulins if not stored immediately at frozen conditions. Thus, other matrices (serum, plasma, etc.) might be preferable in situations precluding immediate postcollection freezing [17]. Generally, also quantification of insulin and C-peptide might be possible with the present assay but requires further characterization of the method. At present, the simultaneous, semiquantitative determination of C-peptide and insulin enables a fast evaluation of endogenous versus exogenous insulin exposure, including the characterization of the type exogenous insulin and the diagnosis of defective or suppressed insulin production. Although sample collection is much more convenient compared to venipuncture, the analysis of urine for peptide-based drugs represents a challenge due to the variability of the matrix. In contrast to blood (serum or plasma), the urinary matrix is often not well defined and parameters such as pH, density, protein content, particulate, etc. vary considerably. Each of these parameters may affect the quality of the analysis and necessitate careful control, e.g., by monitoring the ISTD, accordingly. In the case of glargine, the assay is focused on the main metabolite [18]. This might be adapted in the case of confirmatory analysis. 

## 3. Materials and Methods

Chemicals such as acetonitrile, glacial acetic acid, dimethylsulfoxide, formic acid, methanol, ammonium hydroxide, porcine insulin, bovine insulin, and [[13C6] Leu26, 30]-C-peptide (human) were obtained from Sigma (Schnelldorf, Germany). All aqueous buffers and solutions were prepared in purified water (MilliQ quality, Frankfurt, Germany). Recombinant human insulin was obtained from Aventis (Frankfurt, Germany). The labeled insulin (internal) standard [[2H10] LeuB6, B11, B15, B17]-insulin (human) was purchased from PeptaNova (Sandhausen, Germany). The used solid-phase extraction cartridges OASIS MCX (30 mg, 3 mL) were from Waters (Eschborn, Germany), and the synthetic insulin analogs lispro (Humalog), aspart (Novorapid), glulisine (Apidra), and insulin degludec (Tresiba) were supplied by Eli Lilly (Indianapolis, IN, USA), Novo Nordisk (Princeton, NJ, USA), and Aventis (Kansas City, MO, USA), respectively. The glargine metabolite (DesB31-32 glargine) was obtained from IBA (Warsaw, Poland).

### 3.1. Urine Samples

Spontaneous urine samples were collected from healthy volunteers (five male, five female), without any known medication prior to sampling, and stored frozen until analysis. These samples were used for the validation (specificity). Here, written consent and approval from the local ethical committee were obtained (No.: 047/2021). Additionally, spontaneous urine samples from patients with type 1 or 2 diabetes using different synthetic insulins (degludec, aspart, and glargine) as part of their usual care were analyzed. Applied doses varied from 18 to 44 units/day, and the samples were collected as spontaneous urine within routine diagnostics. All patients provided written informed consent for further use of their specimens. For this study, 3 mL of urine was used per analysis; the volume might be adapted if more or less sensitivity is required. The urine samples were not treated with any protease inhibitors. 

### 3.2. Sample Preparation Mixed-Cation Exchange

To 3 mL of urine, 10 µL of internal standard solution (containing 0.5 µg/mL labeled insulin and 10 µg/mL labeled C-peptide), 1 mL of a mixture of acetonitrile/methanol (1/1, ice cold), and 50 µL of glacial acetic acid was added. After a short vortex, the sample was centrifuged for 10 min at 4000× *g*, and the supernatant was transferred to a new 10 mL polypropylene tube. Solid-phase extraction was performed with mixed-mode cation-exchange cartridges (Waters MCX, 3 cm, 30 mg), which were preconditioned with 1 mL of methanol and 1 mL of water prior to load with the sample solution in two steps. The samples were washed with 2 mL of water and 2 mL of methanol (acidified with 2% of acetic acid; this mixture is freshly prepared every working day). Finally, the sample was eluted into a new 1.5 mL Eppendorf tube with 1.2 mL of a mixture of methanol/ammonium hydroxide solution (5:1), and the volume was reduced to near dryness in a vacuum centrifuge. The residue was diluted with 80 µL of aqueous acetic acid (1%), and 15–25 µL were injected into the LC-MS system.

### 3.3. Liquid Chromatography 

Chromatographic separation of the target analytes was conducted by high-performance liquid chromatography using a Vanquish system (Thermo, Bremen, Germany). The system was equipped with a dual-pump setup and initial trapping of the target analytes on a short trapping column Accucore Phenyl/Hexyl, 3 × 10 mm, 2.7 µm PS (Thermo, Bremen, Germany) using a water solution of formic acid (0.1%, solvent A1) and acetonitrile (with 0.1% formic acid, solvent B1). Trapping was performed for 2 min at 99% of solvent A1 before switching the flow to the analytical column. Since solvent buffers A2 and B2 for generating the gradient, water solution of formic acid (0.1%, with 1% of DMSO, solvent A2) and acetonitrile with DMSO (1%), and water solution of formic acid (0.1%) (solvent B2) were used. As analytical column, a Poroshell C18 3 × 50 mm (Agilent, Karlsruhe, Germany) was utilized, and the flow was set to 400 µL/min. The gradient started at 99% A2 and decreased to 40% A2 within 8 min. Within the next 2 min, the gradient decreased to 20% A2 for cleaning the column. Finally, the system was reequilibrated for 5 min at starting conditions. The resulting overall run time was 15 min, and the injection volume was 15–25 µL. The column compartment was set to 25 °C. 

### 3.4. Mass Spectrometry

An Orbitrap Exploris 480 high-resolution mass spectrometer (Thermo, Bremen, Germany), equipped with a heated electrospray ion source, was used for the detection of the target peptides. The instrument was operated in positive ionization mode acquiring data in full scan mode (*m/z* = 400–1700, resolution 60,000 FWHM) and targeted MS2 by means of an inclusion list. Targeted MS2 (PRM) experiments were performed for the multiply protonated molecules of the target peptides and multiplexed four times with a quadrupole isolation window of 2 *m/z* units. The targeted MS2 experiments were acquired with a resolution of 45,000 FWHM. Multiplex groups were defined as follows: Group 1: aspart, glulisine, glargine metabolite; group 2: lispro, bovine, degludec; group 3: porcine, C-peptide; group 4: ISTD insulin and ISTD C-peptide. For all insulin analogs, the respective five- and fourfold protonated precursor ions were used for MS2 experiments (except degludec: fourfold protonated precursor only). For C-peptide and the respective ISTD, the doubly charged precursor ion was used. The corresponding diagnostic product ions are listed in Table 1. The instrument was calibrated according to the manufacturer‘s recommendations using a calibration mixture (consisting of caffeine, the tetrapeptide MRFA, and Ultramark). The gas supply consisted of nitrogen (N2-generator, CMC, Eschborn, Germany). Ionization in positive mode was accomplished at a voltage of 3 kV, and the temperature of the ion transfer tube was adjusted to 320 °C. Xcalibur Software used was: Foundation 3.1SP7 QF1, Xcalibur 4.4 (Thermo, Bremen, Germany) Main characteristics of the target analytes are summarized in Table 1.

### 3.5. Validation

The validation of the assay was performed under consideration of qualitative result interpretation and the requirements of the WADA (ISL 11) including the parameters specificity, limit of detection (LOD), limit of identification (LOI), linearity, recovery, carryover, matrix effects, and robustness [16]. Urine from healthy volunteers (for all synthetic analogs) and from a patient with *longstanding type 1 diabetes mellitus* (for human insulin and C-Peptide) were used.

#### 3.5.1. Specificity

The specificity of the method was demonstrated by 10 blank urine samples from 10 healthy volunteers (five male, five female), who were proven not to receive a therapeutic insulin regimen. 

#### 3.5.2. Recovery

In order to demonstrate the recovery of target analytes during processing samples were prepared as follows. Six blank urine samples were spiked at 100 pg/mL of all target analytes before processing and six further samples were spiked after processing prior injection into the LC–MS. With the comparison of the results, the recovery of the method was calculated in percent. The recovery for human insulin and C-peptide was determined with a urine sample from a person with type 1 diabetes.

#### 3.5.3. Limit of Detection/Limit of Identification (LOD/LOI)

The limit of detection was determined in a representative blank urine sample fortified with 0, 5, 10, 25, and 50 pg/mL of all target analytes. The signal-to-noise ratio (S/N) at the respective retention time was evaluated for all target analytes. Noteworthy, for evaluation of the LOD for human insulin and C-Peptide, a urine sample from a person with type 1 diabetes was used. The limit of identification (LOI) was calculated by fortifying six different urine samples (from healthy volunteers) at 5, 10, 25, and 50 pg/mL (corresponding to 10, 20, 50, and 100% of the MRPL) with all synthetic insulin analogs. 

#### 3.5.4. Precision

Relative standard deviations of the peak area ratios from a sixfold determination of fortified sample aliquots (at 100 pg/mL) were calculated to show the precision of the assay.

#### 3.5.5. Carry-Over

A possible carryover of analyte from one sample to the next was investigated by injecting a blank sample after a spiked sample from the upper working range (500 pg/mL) and inspecting the respective chromatograms for possible carryover signals. 

#### 3.5.6. Matrix Effect

The comparison of the signal intensities of five processed blank urine samples that were subsequently spiked with all target analytes with a neat standard aliquot that does not contain any matrix is used to determine the matrix effects.

#### 3.5.7. Robustness

One of the critical steps is the degradation of the peptides in the vacuum centrifuge; thus, the impact of the drying time was evaluated for the different peptides. Therefore, six fortified sample aliquots (spiked at 100 pg/mL) were evaporated in the vacuum centrifuge to near dryness (two aliquots), to dryness (two aliquots), and to dryness plus 15 min (two aliquots).

## 4. Conclusions

In summary, the method shown here can be used for simplified qualitative determination of insulin and its synthetic or animal analogs and C-peptide in urine samples. Due to its noninvasive and convenient sample collection, the method may open new diagnostic avenues, especially in the field of pediatrics. The method fulfills the criteria for doping control analysis considering the MRPL of 50 pg/mL while allowing for omission of immunoaffinity purification steps, which provides a considerable benefit in terms of sample preparation efficiency for the initial testing and the majority of samples are tested negative. Nevertheless, in the case of confirmatory analysis in doping controls, applying the additionally established immunoaffinity-based LC–MS test methods is strongly recommended due to commonly superior LODs and enhanced specificity. This is especially true for samples with unknown and uncontrolled backgrounds/origins (such as doping control samples or forensic cases). 

## Figures and Tables

**Table 1:** Main characteristics of all target analytes. Modifications in the amino acid sequence, the protonated molecular ion (precursor) with charge state, and most abundant product ions (with type as subscription) are in bold. (* stable isotope-labeled amino acids).

Peptides Included	Amino Acid Sequence	Monoisotopic Mass [Da]	Precursor Ion [*m/z*]	Monitored Charge State	Product Ions	Multiple × Group	~Ret. Time [min]
Human insulin	GIVEQCCTSICSLYQLENYCN—FVNQHLCGSHLVEALYLVCGERGFFYTPKT	5803.6	1452/1162	4+/5+	**226_y_**, 219_b_, 143_a_, 345_y_	2	7.05
Insulin aspart	GIVEQCCTSICSLYQLENYCN—FVNQHLCGSHLVEALYLVCGERGFFYT**D**KT	5821.6	1457/1166	4+/5+	**226_y_**, **219_b_**, 248_y_, 464_y_	1	7.02
Insulin glulisine	GIVEQCCTSICSLYQLENYCN—FV**K**QHLCGSHLVEALYLVCGERGFFYTP**E**T	5818.6	1456/1166	4+/5+	**227_y_**, **346_y_**, 199_y_	1	7.01
Insulin lispro	GIVEQCCTSICSLYQLENYCN—FVNQHLCGSHLVEALYLVCGERGFFYT**K*P***T	5803.6	1452/1162	4+/5+	**217_y_**, 230_y_	2	7.01
Insulin glargine met	GIVEQCCTSICSLYQLENYC**G**—FVNQHLCGSHLVEALYLVCGERGFFYTPKT	5746.6	1438 /1151	4+/5+	**226_y_**, 219_b_	1	7.09
Insulin degludec	GIVEQCCTSICSLYQLENYCN—FVNQHLCGSHLVEALYLVCGERGFFYTPK-**γ**-**L**-**Glu**-**Pal**	6099.8	1527	4+	**641_y_**, 244_y_	2	8.00
Porcine insulin	GIVEQCCTSICSLYQLENYCN—FVNQHLCGSHLVEALYLVCGERGFFYTPK**A**	5773.6	1445/1156	4+/5+	**226_y_**, 315_y_	3	7.06
Bovine insulin	GIVEQCC**A**S**V**CSLYQLENYCN—FVNQHLCGSHLVEALYLVCGERGFFYTPK**A**	5729.6	1433/1147	4+/5+	**226_y_**, 315_y_	2	6.99
C-peptide	EAEDLQVGQVELGGGPGAGSLQPLALEGSLQ	3018.5	1510	2+	**927**, 260_y_, 785_b_	3	7.22
labeled insulin	GIVEQCCTSICSLYQLENYCN—FVNQHL*CGSHL*VEAL*YL*VCGERGFFYTPKT	5843.9	1462/1170	4+/5+	**226_y_**, 219_b_, 143_a_, 345_y_	4	7.01
labeled C-peptide	EAEDLQVGQVELGGGPGAGSLQPLAL*EGSL*Q	3030.6	1516	2+	**939_y_**, 266_y_, 785_b_	4	7.21

**Table 2:** Main validation results for all target analytes.

	Specificity	LOD	LOI				Precision	Recovery	Carry Over	Matrix Effect
		[pg/mL]	At 50 pg/mL	At 25 pg/mL	At 10 pg/mL	At 5 pg/mL	At 100 pg/mL [%]	[%]	[%]	[%]
Human insulin	ok	10	-	-	-	-	2	35	<1%	-
Insulin aspart	ok	10	6/6	6/6	4/6	3/6	9	26	<1%	80–120
Insulin glulisine	ok	10	6/6	6/6	4/6	3/6	8	26	<2%	80–120
Insulin lispro	ok	10	6/6	6/6	5/6	3/6	8	31	<2%	80–120
Insulin glargine met	ok	10	6/6	6/6	4/6	2/6	13	39	<1%	80–120
Insulin degludec	ok	10	6/6	6/6	5/6	4/6	24	51	<2%	80–120
Porcine Insulin	ok	25	6/6	4/6	2/6	0/6	9	34	<2%	80–120
Bovine Insulin	ok	10	6/6	6/6	5/6	4/6	10	35	<1%	80–120
C-peptide	ok	25	-	-	-	-	3	100	<2%	-

**Table 3:** Main characteristics and results for the postadministration sample analysis.

Patient Number	Diabetes Type	Insulin Regimen	Detected Peptides
1	II	Tujon (glargine)	glargine metabolite, human insulin, C-peptide
2	I	Fiasp (aspart), 44 U/d	aspart
3	II	Fiasp (aspart), tiny doses	aspart, human insulin, C-peptide
4	unclear	Novorapid, 24 U/d; Lantus 24 U/d	aspart, glargine (lantus) metabolite, human insulin, C-peptide
5	unknown	Novorapid, 24 U/d; Tresiba 19 U/d	aspart, tresiba (degludec), C-peptide (traces)

## Data Availability

The data that support the findings of this study are available from the corresponding author upon reasonable request. The data are not publicly available due to large size of the data volume.

## References

[1] Rebsomen L., Pitel S., Boubred F., Buffat C., Feuerstein J.M., Raccah D., Vague P., Tsimaratos M. (2006). C-peptide replacement improves weight gain and renal function in diabetic rats. Diabetes Metab..

[2] Lebowitz M.R., Blumenthal S.A. (1993). The molar ratio of insulin to c-peptide. An aid to the diagnosis of hypoglycemia due to surreptitious (or inadvertent) insulin administration. Arch. Intern. Med..

[3] Blackburn M. (2013). Advances in the quantitation of therapeutic insulin analogues by lc–ms/ms. Bioanalysis.

[4] Cobelli C., Pacini G. (1988). Insulin secretion and hepatic extraction in humans by minimal modeling of c-peptide and insulin kinetics. Diabetes.

[5] (2019). WADA. https://www.Wada-ama.Org/sites/default/files/resources/files/td2019mrpl_eng.Pdf.

[6] Judak P., Coppieters G., Lapauw B., Van Eenoo P., Deventer K. (2020). Urinary detection of rapid-acting insulin analogs in healthy humans. Drug Test Anal..

[7] Thomas A., Thevis M., Makowski G.S. (2019). Recent advances in the determination of insulins from biological fluids. Advances in Clinical Chemistry.

[8] Mazzarino M., Senofonte M., Martinelli F., de la Torre X., Botre F. (2019). Detection of recombinant insulins in human urine by liquid chromatography-electrospray ionization tandem mass spectrometry after immunoaffinity purification based on monolithic microcolumns. Anal. Bioanal. Chem..

[9] Judak P., Van Eenoo P., Deventer K. (2018). Utilizing elisa-plate based immunopurification and liquid chromatography-tandem mass spectrometry for the urinary detection of short- and long acting human insulin analogues. J. Pharm. Biomed. Anal..

[10] Thomas A., Schänzer W., Thevis M. (2017). Immunoaffinity techniques coupled to mass spectrometry for the analysis of human peptide hormones: Advances and applications. Expert Rev. Proteom..

[11] Judak P., Van Eenoo P., Deventer K. (2017). Adsorption effects of the doping relevant peptides insulin lispro, synachten, tb-500 and ghrp 5. Anal. Biochem..

[12] Thomas A., Schänzer W., Delahaut P., Thevis M. (2012). Immunoaffinity purification of peptide hormones prior to liquid chromatography-mass spectrometry in doping controls. Methods.

[13] Thomas A., Yang R., Petring S., Bally L., Thevis M. (2020). Simplified quantification of insulin, its synthetic analogs and c-peptide in human plasma by means of lc-hrms. Drug Test. Anal..

[14] Chambers E.E., Fountain K.J., Smith N., Ashraf L., Karalliedde J., Cowan D., Legido-Quigley C. (2014). Multidimensional lc-ms/ms enables simultaneous quantification of intact human insulin and five recombinant analogs in human plasma. Anal. Chem..

[15] Thomas A., Thevis M., Delahaut P., Bosseloir A., Schanzer W. (2007). Mass spectrometric identification of degradation products of insulin and its long-acting analogues in human urine for doping control purposes. Anal. Chem..

[16] (2021). WADA. https://www.Wada-ama.Org/en/resources/laboratories/international-standard-for-laboratories-isl.

[17] Wunder C., Kauert G.F., Toennes S.W. (2014). Factors leading to the degradation/loss of insulin in postmortem blood samples. Forensic Sci. Int..

[18] Kuerzel G.U., Shukla U., Scholtz H.E., Pretorius S.G., Wessels D.H., Venter C., Potgieter M.A., Lang A.M., Koose T., Bernhardt E. (2003). Biotransformation of insulin glargine after subcutaneous injection in healthy subjects. Curr. Med. Res. Opin..

